# Polariton nanophotonics using phase-change materials

**DOI:** 10.1038/s41467-019-12439-4

**Published:** 2019-10-03

**Authors:** Kundan Chaudhary, Michele Tamagnone, Xinghui Yin, Christina M. Spägele, Stefano L. Oscurato, Jiahan Li, Christoph Persch, Ruoping Li, Noah A. Rubin, Luis A. Jauregui, Kenji Watanabe, Takashi Taniguchi, Philip Kim, Matthias Wuttig, James H. Edgar, Antonio Ambrosio, Federico Capasso

**Affiliations:** 1 Harvard John A. Paulson School of Engineering and Applied Sciences, Harvard University, Cambridge, MA 02138 USA; 2 Department of Physics “E. Pancini”, University of Naples “Federico II”, Complesso Universitario di Monte S. Angelo, Via Cinthia 21, 80126 Naples, Italy; 3 Department of Chemical Engineering, Kansas State University, Manhattan, KS 66506 USA; 4 1. Physikalisches Institut IA, RWTH Aachen University, 52056 Aachen, Germany; 5 Department of Physics and Astronomy, University of California, Irvine, CA 92697 USA; 6 National Institute for Materials Science, 1-1 Namiki, Tsukuba, 305-0044 Japan; 7 Department of Physics, Harvard University, Cambridge, MA 02138 USA; 8 Center for Nanoscale Systems, Harvard University, Cambridge, MA 02138 USA; 9 CNST – Fondazione Istituto Italiano di Tecnologia, Via Pascoli 70/3, 20133 Milano, Italy

**Keywords:** Nanoscale devices, Optical materials and structures, Polaritons, Sub-wavelength optics

## Abstract

Polaritons formed by the coupling of light and material excitations enable light-matter interactions at the nanoscale beyond what is currently possible with conventional optics. However, novel techniques are required to control the propagation of polaritons at the nanoscale and to implement the first practical devices. Here we report the experimental realization of polariton refractive and meta-optics in the mid-infrared by exploiting the properties of low-loss phonon polaritons in isotopically pure hexagonal boron nitride interacting with the surrounding dielectric environment comprising the low-loss phase change material Ge_3_Sb_2_Te_6_. We demonstrate rewritable waveguides, refractive optical elements such as lenses, prisms, and metalenses, which allow for polariton wavefront engineering and sub-wavelength focusing. This method will enable the realization of programmable miniaturized integrated optoelectronic devices and on-demand biosensors based on high quality phonon resonators.

## Abstract (web summary)

Here, the authors experimentally demonstrate a platform for tunable polariton refractive and meta-optics based on hexagonal boron nitride and phase change Ge_3_Sb_2_Te_6_. This combination has the advantage of the long-lived phonon-polariton with switchable refractive index of the phase change material.

## Introduction

Polaritons in van der Waals materials behave as confined guided optical modes, which extend as evanescent waves into the semi-spaces above and below^1–19^. Therefore, their propagation is affected by the refractive indices of the superstrate and substrate^3,4,6,8^. One notable example of these polaritons are phonon polaritons (PhPs) in hexagonal boron nitride (hBN). These are not interface modes as for plasmons in noble metals: they exist instead inside the volume of hBN. The permittivity values are of opposite signs along different principal axes and thus polaritons exhibit hyperbolic dispersion^3,8,12^. The degree to which the optical energy density of polaritons extends into the substrate and superstrate depends on the wavelength and thickness of hBN. Therefore, the excitation wavelength can be controlled to the point where the polariton is affected even by the very first few nanometers of the substrate and superstrate^18^. This suggests the feasibility of substrate-engineered polariton optics where, instead of nanopatterning the polaritonic material itself, optical functions such as waveguiding and focusing are conferred through engineering the refractive index of the substrate.

A heterostructure comprising the phase-change material Ge_3_Sb_2_Te_6_ (GST) and isotopically pure h^11^BN (referred to hBN hereinafter) is the ideal system for a proof-of-concept demonstration of substrate-engineered polariton optics: hBN possesses low-loss polaritons with long propagation lengths^5^ and GST can support two vastly different refractive indices in its amorphous and crystalline phases (*n*_a_ = 4.2 and *n*_c_ = 6.1), which can co-exist at room temperature^20–29^. While GST has been previously employed to demonstrate switchable PhP resonators in quartz^27^, more complex applications including metasurfaces remained elusive due to limited propagation lengths of PhPs in quartz. On the other hand, *V*O_2_ has been used as a substrate with hBN to achieve temperature-dependent polariton dispersion^28^. However, this approach suffers from limitations because *V*O_2_’s different phases cannot co-exist at the same temperature, and thus in close spatial proximity as is desirable for the realisation of optical devices. Here, we show an hBN-GST heterostructure in which arbitrary patterns can be written, erased, and re-written to control the PhP propagation. We achieve this by defining several structures, ranging from waveguides^30,31^ to diffraction-limited focusing metalenses. Specifically, we use low-loss PhPs in isotopically pure hBN (^11^B isotopes with >99% purity^5^) with longer propagation lengths, which we combine with Ge_3_Sb_2_Te_6_, a stoichiometry with particularly low absorption in the mid-infrared (mid-IR)^29^. We work in the second Reststrahlen band (from 1361–1610 cm^−1^), which is associated with in-plane optical phonons^5,6^.

## Results

### Reconfigurable 2D refractive polariton lenses

**Fig. 1:**
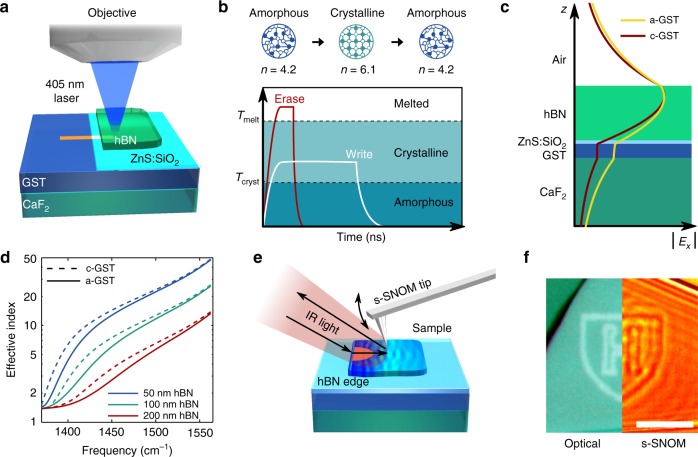
Reconfigurable polaritons in hBN-GST heterostructures. **a** Writing setup and device cross-section. A 405 nm focused laser beam is used to write and reconfigure devices on GST underneath hBN (transparent at 405 nm). **b** Longer, low-power laser pulses are used to crystallise GST and shorter high-power pulses are used to melt it to restore the amorphous phase. **c** Electric field profile of polaritons for the a-GST and c-GST cases. The electric field confinement is larger in c-GST (due to its larger refractive index) than in a-GST. *E*_*x*_ represents the electric field along the direction of polariton propagation. Thicknesses for each layer are 195 nm for hBN, 15 nm for ZnS:SiO_2_, 55 nm for GST and 1 mm for CaF_2_, which is then considered semi-infinite. Refractive indices are 1.7 for ZnS:SiO_2_, 4.2 and 6.1 for GST in amorphous and crystalline phases, respectively, 1.37 for CaF_2_, while hBN is modelled with the Lorentz model presented in Supplementary Note 1. **d** Calculated dispersion relation of the effective index *n*_eff_ for different hBN thicknesses on a-GST and c-GST. **e** Polaritons are launched by the hBN edge when light impinges on the sample. The launched polaritons interact with the written devices and their propagation is imaged using scattering-type scanning near field optical microscopy (s-SNOM). **f** Example of optical and s-SNOM images. Scale bar is 5 µm

Figure 1a shows the hBN-GST heterostructure used in this work. As PhPs are strongly confined, a thin layer of 55 nm of GST below hBN (195 nm of thickness) is sufficient to significantly alter polariton propagation. To create the heterostructure, we sputter a thin film of GST on a CaF_2_ substrate (in an amorphous phase as-deposited), protect it with a 15 nm layer of ZnS:SiO_2_ against oxidation and then transfer exfoliated hBN to form the top layer. A pulsed laser diode is used to write and erase patterns by selectively crystallising or re-amorphising GST^22^ (Fig. 1b).

The PhP mode profile is affected by the state (either crystalline or amorphous) of the GST beneath it (Fig. 1c), which can be quantified by using the effective index $n_{{\mathrm{eff}}} = c{\mathrm{/}}v_{{\mathrm{ph}}}$, where *c* is the speed of light and *v*_ph_ is the PhP’s phase velocity. Figure 1d shows the dispersion of *n*_eff_ for different hBN thicknesses on both a-GST (*n*_eff,__a_) and c-GST (*n*_eff__,c_), respectively. We use scattering-type scanning near field optical microscopy (s-SNOM) to characterise the polaritons launched at hBN edges, which propagate across the optical elements (Fig. 1e, f)^8,13,18^.

Polariton propagation in heterostructures with a- or c-GST is analogous to light propagation in two different materials (such as air and glass). The continuity of the electric field at the boundary between two regions implies Snell’s law^32^:

1 $$\frac{{n_{{\mathrm{eff}},{\mathrm{c}}}}}{{n_{{\mathrm{eff}},{\mathrm{a}}}}} = \frac{{\sin \left( {\theta _{\mathrm{a}}} \right)}}{{\sin \left( {\theta _{\mathrm{c}}} \right)}},$$

where $\theta$ is the propagation angle in corresponding regions with respect to the interface normal.

**Fig. 2:**
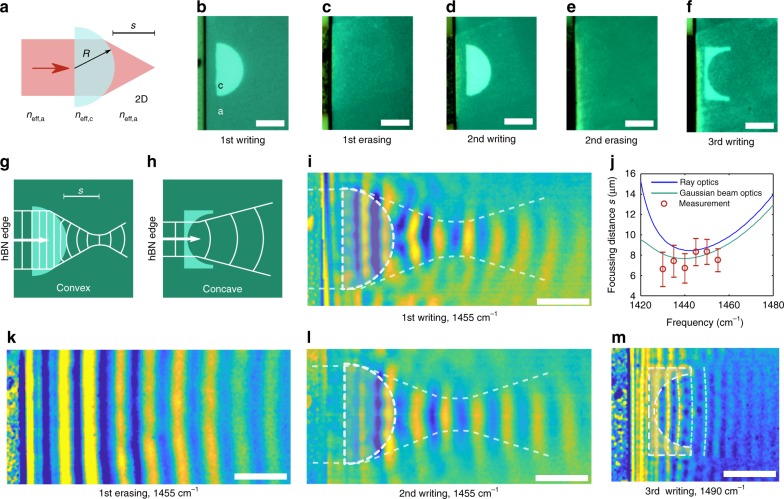
Rewritable flat polaritonic lenses. **a** Plano-convex lens schematics for 3D and 2D semi-spherical and semi-circular lenses. In the 2D case the material refractive index is replaced by the effective index of the polaritons on amorphous or crystalline GST. **b–f** Optical images of the written lens. The written patterns are clearly visible in the pictures because the refractive index of a-GST and c-GST also differs at visible wavelengths. First a plano-convex semi-circular lens (radius *R* = 5 µm) is written and measured, then it is erased, re-written (with same dimensions), erased again and finally the same area is reconfigured into a plano-concave lens (*R* = 5 µm). **g**, **h** Diagram of wavefronts for 2D plano-convex and plano-concave lenses, respectively. **i** s-SNOM image of the plano-convex lens after the first writing. A focal spot is clearly visible. **j** Dependence of the focal length on the wavenumber. Error bars represent the uncertainty of the fitted waist position, approximately one fringe). **k** s-SNOM scan after first erasing. **l** s-SNOM scan of the re-written plano-convex lens. **m** s-SNOM scan of the plano-concave lens (third writing). s-SNOM images in **i**, **l** and **m** have been processed to remove the fringes outside the main beam (see Supplementary Note 4, Supplementary Table 3 and Supplementary Fig. 5). Scale bars are 5 µm

Many conventional optical devices (such as lenses and prisms) are governed by Snell’s law, suggesting that similar components can be implemented in our hBN-GST heterostructure. The first example to illustrate this principle is a refractive lens, specifically, a plano-convex semi-circular lens to focus PhPs (Fig. 2a).

We write and erase a semi-circular plano-convex lens (radius (*R* = 5 μm)) twice and subsequently replace it with a plano-concave lens of the same radius (Fig. 2b–f). The straight hBN edge launches linear waves (the planar equivalent of three-dimensional plane waves), which are either focused by the plano-convex lens or diverged by the plano-concave lens (Fig. 1g, h). The lateral size of the focal spot is 2 μm (29% of the free space wavelength), which is diffraction limited according to the Abbe limit computed for the 2D waves (i.e. 2.08 μm). The numerical aperture (NA) with respect to polaritons in a-GST is 0.55, while the NA with respect to vacuum is 2.11, which is higher than unity due to the high confinement of PhPs.

We performed phase-resolved s-SNOM measurements after each writing and erasing step. Using amplitude and phase information, the wavefronts of the polaritons can be clearly imaged (see Supplementary Note 4 for more details on measurements and image processing). The resulting images confirm focusing, which is shown by the narrow waist in the transmitted beam (Fig. 1i). Characteristic curved wavefronts can be seen before and after the focal spot. The position of the focal spot of the lens is measured from the images and compared with theoretical computation with two different methods (Fig. 2j). The first method is based on computing the focal spot using ray optics, while the second, more accurate, method models the focused beam as a Gaussian beam, and identifies the focal spot as the beam waist^32^ (see more details in the Supplementary Note 2, Supplementary Fig. 1 and Supplementary Table 1).

After erasing, only polaritons with linear wavefronts that are launched by the hBN edge are visible, whereas focusing is fully restored when the lens is re-written (Fig. 2k, l). Furthermore, reconfiguring the same area to a plano-concave lens results in curved wavefronts associated with diverging PhPs (Fig. 2m).

### Prisms and waveguides

**Fig. 3:**
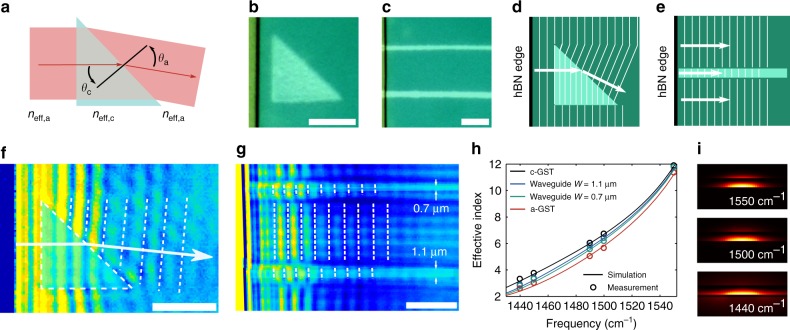
Prism and waveguides. **a** Snell’s law for 2D prisms determines deflection of polaritons. **b** Optical image of the written prism, an isosceles right-angled triangle with edges of 7.5 µm. The flake edge is also visible. **c** Optical image of the written waveguides (top 0.7 µm wide, bottom 1.1 µm wide). The distance between the waveguides is 8.5 µm, which ensures no coupling between them. **d** Diagram of wavefronts for the prism. **e** Schematics of wavefronts for a waveguide. Polaritons propagating inside the waveguide have smaller fringe spacing due to the additional confinement of the waveguide mode. **f** s-SNOM image of prism showing a clear deflection angle of the outgoing wavefronts. **g** s-SNOM image of waveguides, showing the expected confinement of the modes inside of them. The fringe spacings are different for waveguides with different widths, confirming that the spacing is determined by the mode of the waveguide. **h** Simulated and measured effective indices of the waveguides. The effective indices are between n_eff_,_a_ and n_eff_,_c_. **i** Cross-section of the guided mode of the 0.7 µm waveguide at different frequencies (out-of-plane Poynting vector). Scale bars are 5 µm

The successful implementation of lenses can be extended to other common devices such as prisms and waveguides (Fig. 3). Planar prisms are simply triangles and follow Snell’s law (Fig. 3a). We wrote a prism and two waveguides (with different widths) close to the hBN edge so that edge-launched waves can interact with them (Fig. 3b–e). The prism is designed to be an isosceles right triangle with one side parallel to the hBN edge such that edge-launched waves enter orthogonal to it. When traversing the hypotenuse, the polariton propagation direction (***k*** vector) is bent downwards (as expected from Snell’s law), as is clearly visible in the s-SNOM measurements in the form of bent fringes (Fig. 3f).

The waveguides consist of c-GST lines with widths (0.7 and 1.1 µm) smaller or comparable to the guided polariton wavelength. They provide additional in-plane confinement such that the propagating mode is truly one-dimensional and is confined along the waveguide. Here, the c-GST line acts as the waveguide core, while a-GST serves as cladding. The s-SNOM measurement in Fig. 3g shows that the wavefront spacing decreases inside the waveguides, as expected from confined modes. Furthermore, the compression is greater for the wider waveguide. This implies that the waveguide effective index *n*_eff_,_wg_ is larger when the width of the waveguide increases, which agrees with the behaviour known from conventional dielectric waveguides where the core size affects the effective index of the mode. In both the conventional and the polariton cases, the value of the waveguide effective index lies between the indices of the core and cladding material, that is, $n_{{\mathrm{eff}},{\mathrm{a}}} \le n_{{\mathrm{eff}},{\mathrm{wg}}} \le n_{{\mathrm{eff}},{\mathrm{c}}}.$ We verified this behaviour by numerically calculating the waveguide dispersion relation (see Methods) and comparing the results to s-SNOM measurements taken at different frequencies (Fig. 3h). Figure 3i shows a cross-section of a guided mode obtained from numerical simulation, illustrating how polaritons are confined both vertically and laterally.

### Reconfigurable polariton metalenses

**Fig. 4:**
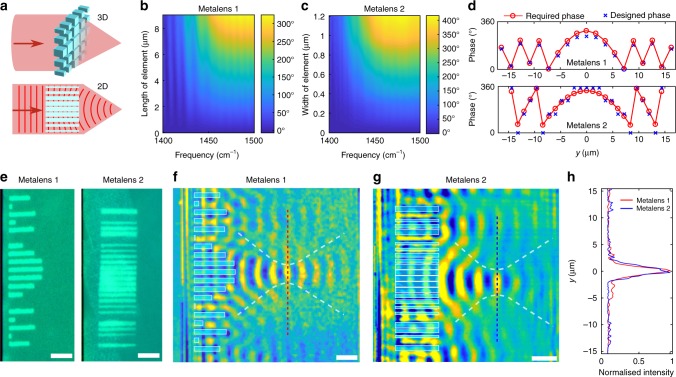
Reconfigurable metalenses. **a** Adaptation of metalenses to the 2D case. Each discrete element changes the local phase of light, so the wavefronts converge to a focal spot. **b**, **c** Local phase (in degrees) of each element as a function of the element parameter and the frequency. **b** refers to unit cells with period 1.8 µm, 1 µm width and variable lengths. **c** refers to unit cells with period 1.2 µm, 9 µm length and variable widths. **d** Designed phase profile of the two metalenses (see Supplementary Note 2 for additional information). The design frequency was 1452 and 1448 cm^−1^ for metalens 1 and metalens 2, respectively. **e** Optical images of the written metalenses. Metalens 1 was written, characterised, erased and subsequently the same area was reconfigured into metalens 2. **f** s-SNOM image of metalens 1 showing focusing of polaritons at 1452 cm^−1^. **g** s-SNOM image of metalens 2 at 1445 cm^−1^. **h** Intensity profile (square of s-SNOM signal) at the cross-section of the diffraction-limited focused polariton beam for both lenses (dashed vertical lines in **f**, **g**). Scale bars are 5 µm

Metasurfaces have recently emerged as a novel and versatile method for engineering light propagation by using arrays of discrete elements, which locally alter the phase of transmitted light. By changing the size and shape of these elements, arbitrary predetermined phase profiles can be implemented^33^. Figure 4 shows the adaptation of this concept for polaritons and its implementation in an hBN-GST heterostructure. Our approach allows designing one-dimensional metalenses, which focus polaritons that propagate in two dimensions. The metalens elements are truncated c-GST waveguides defined in a-GST environment and create the hyperbolic phase profile^34^:

2 $$\phi \left( y \right) = - \frac{{2\pi }}{{\lambda _{{\mathrm{eff}},{\mathrm{a}}}}}\left( {\sqrt {y^2 + f^2} - f} \right),$$

where *y* is the element position in *y*-direction and *f* is the focal length (Fig. 4a). We build a phase library for elements of varying lengths (metalens 1, periodicity of unit cell: 1.8 µm) and widths (metalens 2, periodicity of unit cell: 1.2 µm) (Fig. 4b, c), and subsequently incorporate the required phase profile by choosing the corresponding elements (Fig. 4d)^33^. First, we demonstrate a metalens based on length changes, then we erase and replace it with a metalens where only the width of the individual elements varies. s-SNOM characterisation was carried out after each step and clearly reveals the focusing effect of the two designed lenses (Fig. 4f, g). Figure 4h shows the confinement of polaritons at the focal spot. Quantitative analysis (see Supplementary Table 1) of both metalenses confirms that we were able to achieve spherical aberration-free, diffraction-limited focusing using both approaches: the lateral size of the focal spots are 1.6 and 2 µm, respectively, which is in good agreement with the respective Abbe limits (1.66 and 1.90 µm, respectively). The focal spots are 23% and 29% of the free space wavelength, respectively. The realised phase range is slightly different for the two metalenses. The effect of an incomplete phase range is to have a fraction of the polaritons transmitted without being focused, but the focusing power of the lenses is not affected.

## Discussion

In summary, our results clearly establish that the hBN-GST heterostructure used in this work can serve as a versatile platform to arbitrarily control polaritons at the nanoscale to achieve freeform, transformation, and meta-optics^19^. While we chose to integrate hBN with GST, it can be readily combined with other polaritonic vdW materials^3^ and other phase-change materials^34^, thereby enabling a whole range of deeply sub-wavelength polaritonic devices, from visible to IR spectral regimes. Fully fledged polaritonic circuits can be cheaply fabricated without the need for traditional photolithography, allowing the low-cost realisation of biosensors^15,35^ and high-density optical storage, which benefit from the extreme volume confinement that can be achieved with polaritons. The challenges posed by the propagation losses to the implementation of optical circuits do not prevent the realisation of useful devices. For example, compact polarisers, biosensors and photodetectors could rely on the local enhancement due to resonant polaritons. Alternatively, optimising the layer thickness and materials can reduce the optical energy density inside hBN and therefore the losses. Alternative actuation mechanisms include using electrodes to locally heat GST electrically, as done in commercial GST memories. This approach could also be implemented with graphene, which is compatible with GST^21^. The reconfigurability offered by GST-vdW heterostructures and the possibility of electrically switching GST-vdW material heterostructures paves the way to applications such as modulators, photodetectors and, more generally, programmable optical devices as optical counterparts to field programmable gate arrays.

## Methods

### Sample fabrication

A 55-nm GST-326 film (with 15 nm of ZnS:SiO_2_ protection layer) was sputter coated onto a 1-mm-thick CaF_2_ substrate. Lithography was performed to define alignment markers (positive tone photoresist S1813 spin coated at 3000 RPM and baked at 115 °C for 90 s), followed by Pt sputtering (30 nm) and lift-off at room temperature. Isotopically pure h^11^BN flakes were mechanically exfoliated onto the substrate after plasma activation (5 min of O_2_ plasma at 100 W) using a standard Scotch tape process. We removed traces of the glue from the Scotch tape by placing the sample in acetone for 10 min, followed by an isopropyl alcohol rinse for 5 min and drying with nitrogen. Afterwards, samples were further cleaned with O_2_ plasma (10 min of O_2_ plasma at 100 W). The thicknesses of the flakes and of GST were confirmed through atomic force microscope (AFM) measures (Cypher AFM from Asylum Research).

### Reconfigurable pattern writing

See Supplementary Note 3, Supplementary Figs. 2, 3, 4 and Supplementary Table 2 for details on the technique used to write and erase patterns in GST.

### Lens and metalens parameters

See Supplementary Table 1 for a summary of the fabricated lens parameters.

### Numerical simulations

Numerical simulations were performed using Lumerical Mode solutions and Lumerical FDTD. One-dimensional (1D) simulations in mode solutions were used to calculate the effective indices *n*_eff__,a_, *n*_eff__,c_. A mesh size of 1 nm was used to compute the fundamental mode profile and effective indices of the hBN-GST-326 heterostructures in the RS2 band of h^11^BN. Here, hBN was modelled as an anisotropic dielectric with its permittivity values obtained from the Lorentz model (Supplementary Note 1). The effective indices of the waveguides were calculated via 2D simulations of their cross-sections, also done in Mode Solutions. Metalenses were designed by first simulating numerically each element in a periodic environment with full wave 3D simulations performed in FDTD. The result was a library of elements, which was used to implement the required phase profile. The final metalens was also completely simulated to verify the focusing behaviour. For 3D simulations, we used a mesh size smaller than 50 nm along *x*- and *y*-axis and 5 nm along the *z*-axis. A series of dipole sources were used to excite the polariton modes in hBN in the RS2 band.

### s-SNOM measurements

The near-field scans were obtained using a commercial system from NeaSpec. Tapping-mode AFM is used (tapping amplitude of 130 nm, Pt-Ir-coated tips with resonant frequency of ~300 kHz, tip diameter of ~20 nm). A quantum cascade laser array from Daylight Solutions was used as a tuneable mid-IR source for imaging. The phase-amplitude images are obtained using a pseudo-heterodyne demodulation. The images of lenses and metalenses were processed using the technique shown in the Supplementary Note 4 to isolate the polaritons focused by the structure. In all cases, polaritons are self-launched by the edges^36^. See Supplementary Note 5 and Supplementary Figs. 6, 7, 8 for additional measurements.

## Supplementary information

Supplementary Information

Supplementary Movie 1

Supplementary Movie 2

Supplementary Movie 3

Description of Additional Supplementary Files

## Data Availability

All data are available from the authors upon request. Please contact M.T. for any request of materials and data.
