# Supplementary material for: Polariton nanophotonics using phase-change materials
Source: Nat Commun. 2019 Oct 3;10:4487. doi: 10.1038/s41467-019-12439-4 (PMC6776658; doi:10.1038/s41467-019-12439-4)
Supplement: Supplementary file 1 — Supplementary Information [file 41467_2019_12439_MOESM1_ESM.pdf]

Supplementary Information for "Polariton Nanophotonics using Phase Change Materials"

Kundan Chaudhary[1‡], Michele Tamagnone[1‡*], Xinghui Yin[1‡*], Christina M. Spägele[1‡], Stefano L. Oscurato[1,2], Jiahan Li[3], Christoph Persch[4], Ruoping Li[1], Noah A. Rubin[1], Luis A. Jauregui[5], Kenji Watanabe[6], Takashi Taniguchi[6], Philip Kim[7], Matthias Wuttig[4], James H. Edgar[3], Antonio Ambrosio[8, 9], Federico Capasso[1*]

[1] *Harvard John A. Paulson School of Engineering and Applied Sciences, Harvard University, Cambridge, Massachusetts 02138, USA.*
[2]*Department of Physics "E. Pancini", University of Naples "Federico II", Complesso Universitario di Monte S. Angelo, Via Cinthia 21, 80126, Naples, Italy.*
[3] *Department of Chemical Engineering, Kansas State University, Manhattan, Kansas 66506, USA.*
[4] *1. Physikalisches Institut IA, RWTH Aachen University, 52056 Aachen, Germany.*
[5] *Department of Physics and Astronomy, University of California, Irvine, California 92697, USA*
[6] *National Institute for Materials Science, 1-1 Namiki, Tsukuba, 305-0044, Japan*
[7] *Department of Physics, Harvard University, Cambridge, Massachusetts 02138, USA.*
[8] *Center for Nanoscale Systems, Harvard University, Cambridge, MA 02138, USA.*
[9] *CNST - Fondazione Istituto Italiano di Tecnologia, Via Pascoli 70/3, 20133 Milano, Italy.*
**Correspondence to: mtamagnone@seas.harvard.edu, xyin@seas.harvard.edu, capasso@seas.harvard.edu.*
‡These authors contributed equally to this work.

Supplementary Note 1: isotopically pure hBN permittivity model
The used model for the permittivities is described in Ref. 5:

$$\varepsilon_x = \varepsilon_y = \varepsilon_\perp = \varepsilon_{\infty\perp}\left(1 - \frac{(\omega_{\mathrm{LO},\perp})^2 - (\omega_{\mathrm{TO},\perp})^2}{\omega^2 - j\omega\Gamma_\perp - (\omega_{\mathrm{TO},\perp})^2}\right) \quad \text{(S1)}$$

$$\varepsilon_z = \varepsilon_\parallel = \varepsilon_{\infty\parallel}\left(1 - \frac{(\omega_{\mathrm{LO},\parallel})^2 - (\omega_{\mathrm{TO},\parallel})^2}{\omega^2 - j\omega\Gamma_\parallel - (\omega_{\mathrm{TO},\parallel})^2}\right) \quad \text{(S2)}$$

where the parameters are given in Ref 5 as follow:
In-plane response: $\varepsilon_{\infty\perp} = 5.32$, $\omega_{\mathrm{TO},\perp} = 1359.8\ \mathrm{cm}^{-1}$, $\omega_{\mathrm{LO},\perp} = 1608.7\ \mathrm{cm}^{-1}$, $\Gamma_\perp = 2.1$
Out-of-plane response: $\varepsilon_{\infty\parallel} = 3.15$, $\omega_{\mathrm{TO},\parallel} = 755\ \mathrm{cm}^{-1}$, $\omega_{\mathrm{LO},\parallel} = 814\ \mathrm{cm}^{-1}$, $\Gamma_\parallel = 1$

Supplementary Note 2: Lens and metalens design and analysis
The focus of the fabricated plano-convex lenses cannot be computed with the lens-maker's equation, since these lenses cannot be approximated with thin lenses. Therefore, the focal spot is computed by approximating the lens with a hyperbolic lens which has a well-defined analytical formula for the focal spot position (Supplementary Figure 1). The hyperbolic profile has the equation:

$$(n^2-1)x^2 - y^2 - 2f(n-1)x = 0 \quad \text{(S3)}$$

where $n = n_{\mathrm{eff,c}}/n_{\mathrm{eff,a}}$ is known and f (i.e. the focal length) is fitted. The focal position found in this way strictly relies on ray optics, which is used to derive the hyperbolic profile in the first place.

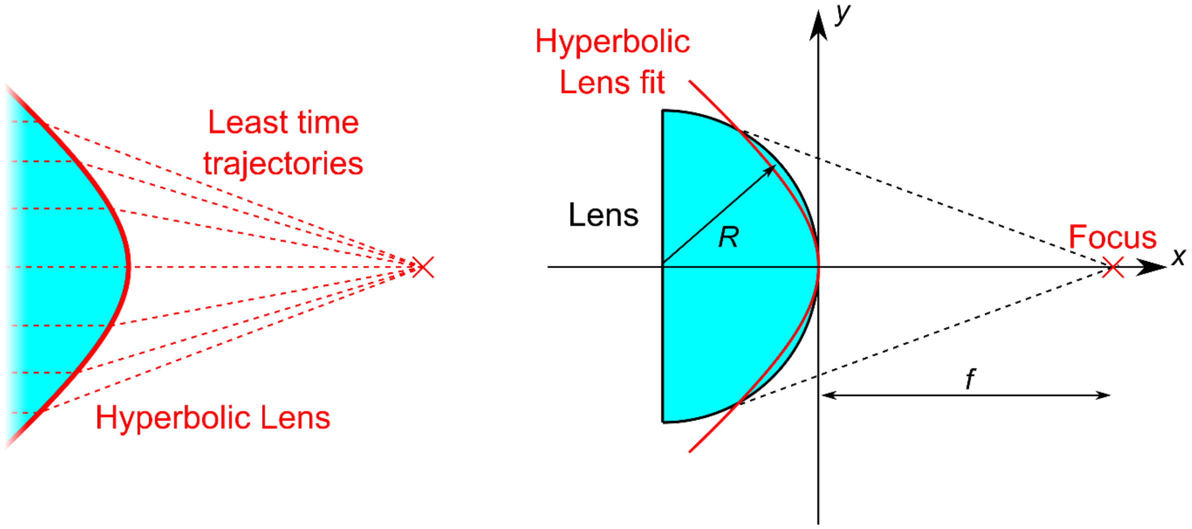

Supplementary Figure 1 | Fitting semi-circular lenses with hyperbolic profile. The hyperbolic lens is the exact locus of points that can focus an incoming plane wave in a single point according to geometric optics. The written lens is semi-circular and is fitted by the hyperbolic profile using *f* as the fitting parameter.

A better description of the focal spot can be obtained by considering that the guided wavelength is comparable to the size of the lens ($R \approx \lambda$) and using gaussian optics. According to gaussian optics, the actual beam waist is at a position *s* (where the focused beam has the smallest lateral size) which is not equal to *f*. Such an equality holds only in the ray optics limit ($R \gg \lambda$). Following ref. 32:

$$s = \frac{Z_{\mathrm{R}}^2 f}{Z_{\mathrm{R}}^2 + f^2} \qquad \text{with} \qquad Z_{\mathrm{R}} = \frac{\pi R^2}{\lambda_a} \tag{S4}$$

where Z_{R} is the Rayleigh range of the input beam defined using the radius R of the lens as the input beam waist and λ_{a} is the SPhP wavelength on amorphous GST ($\lambda_{\mathrm{a}} = \lambda / n_{\mathrm{eff,a}}$). As expected, for the large lens limit, $s = f$ as expected for ray optics.

For the metalens design, each truncated waveguide element is simulated first in a periodic environment as usually done for metasurface design. The phase delay is found relative to the case where no element is present (length or width equal to zero) and then elements are chosen from the desired phase profile. The differences between the design phase and the desired phase profile in Figure 4d are due to discrepancies in the initial estimation of the layer thicknesses (fixed in the last metasurface) and to the phase discretization of the width in the second metasurface, due to the way the waveguides are written (with several passes at a given distance from each other). Supplementary table 1 summarizes the information on the design of the various lenses.

Supplementary Table 1 | Summary of lenses parameters

Property	Semi-circular lens	Metalens 1	Metalens 2
Design wavenumber (cm^{-1})	-	1450	1450
Image wavenumber (cm^{-1})	1455	1452	1448
$n_{\mathrm{eff,a}}$ at image wavenumber	3.04	2.90	2.72
Lateral size of lens (µm)	10	34	28
Focal length at image wavenumber (µm)	7.5	16	15
Numerical aperture w.r.t polariton	0.55	0.73	0.68
Numerical aperture w.r.t vacuum	2.11	1.86	3.04
Lateral spot size, measured (µm)	2	1.6	2
Lateral spot size, Abbe limit (µm)	2.08	1.66	1.90

Supplementary Note 3: Direct laser writing

The patterning of the GST film was carried out using a 405 nm laser diode (disassembled from a Pioneer 16X BDR-209 Blu-Ray writer), which was coupled into a single mode fibre and focused onto the sample through a 0.9 NA objective (Supplementary Figure 2).

The laser diode was driven using an Agilent 8114A pulse generator. All structures were written with 50 ns pulse trains at 17 MHz frequency and a beam moving velocity of 1 µm/s. The pulse peak power used for writing ranged from 3.55 mW to 12.2 mW (details for different structures in Supplementary Table 2). For erasing, shorter pulses of 20 ns duration, 17 MHz repetition rate and a peak power of 28 mW were used. The GST film is ablated outside of the hBN covered regions, which were only protected by a 15 nm layer of $ZnS:SiO_2$ to prevent oxidation of the GST film (Supplementary Figure 3). Typically, one would use a layer thickness on the order of 100 nm to carry out the re-write procedure on GST without hBN. Since we do not observe ablating in regions covered by hBN, we conclude that it serves as excellent capping layer for GST by itself. Furthermore, we observed small specks of crystalline GST after erasing in an optical microscope (Nikon) with 150x magnification, which we couldn't resolve during the erasing with the self-built microscope that we used for monitoring the quality of the erasing. We confirmed however, that these small impurities did not affect polariton propagation.

Given that the necessary powers were very low compared to what is in principle available for the laser diode we used (1000 mW), all demonstrated patterns can be achieved by parallel illumination using a spatial light modulator. In this scenario, the amorphization would not suffer from residual crystalline patches as the

illumination would be homogenous over a large area. Supplementary Figure 4 is an overview of the hBN flake in its final state.

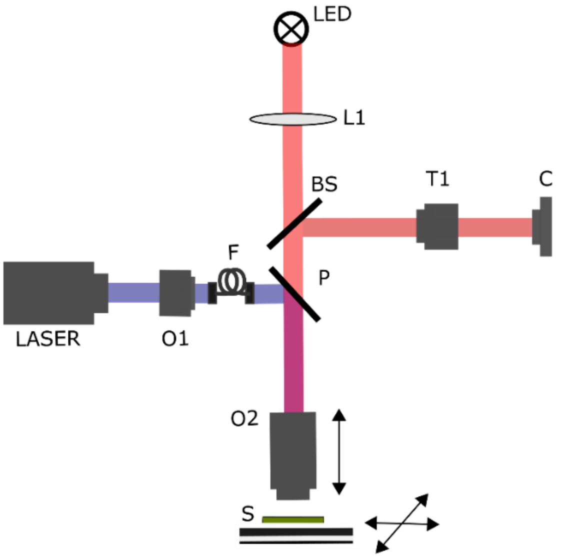

Supplementary Figure 2 | Optical beam path of writing setup. A laser diode operating at 405 nm is focused into a single mode optical fibre and subsequently focused onto the sample through a 0.9 NA objective (O2). The sample is mounted on a programmable stage and the writing is monitored through a microscope consisting of objective O2 and tube lens T1. The writing and microscope arms are combined using a dichroic mirror (P).

Supplementary Table 2 | Writing parameters. The different structures were written and erased at varying peak powers and pulse durations. The frequencies of all writing and erasing processes were 17 MHz and 130 Hz respectively. The beam moving velocity was always 1 µm/s.

Structure	Pulse duration (ns)	Peak Power (mW)
Metalens	50	8.3
Prism	50	3.55
Waveguide 1.1 µm	50	9.3
Waveguide 0.7 µm	50	5.1
Metalens 1 & 2	50	4.1
Plano-convex lens 1 & 2	50	3.7
Plano-concave lens	50	3.7
(Erasing)	20	28

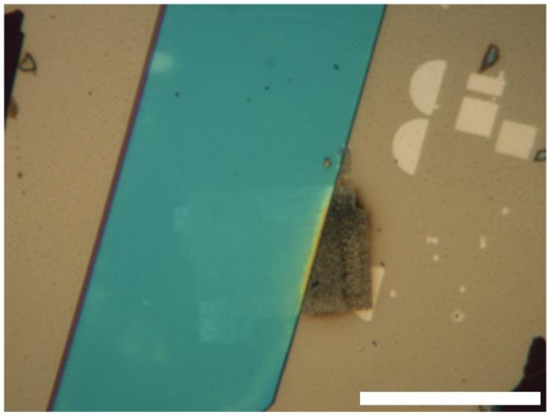

Supplementary Figure 3 | Ablated GST and small crystalline spots. The regions uncovered by hBN are ablated by the high laser powers needed for re-amorphizing, the image shows the region after the second erasure of the refractive lens (just before writing the concave lens). The hBN covered regions are protected by the hBN itself making it an ideal capping layer for GST. The erased region appears slightly brighter than the surrounding amorphous region, which did not affect polariton propagation. The scale bar is 30 µm.

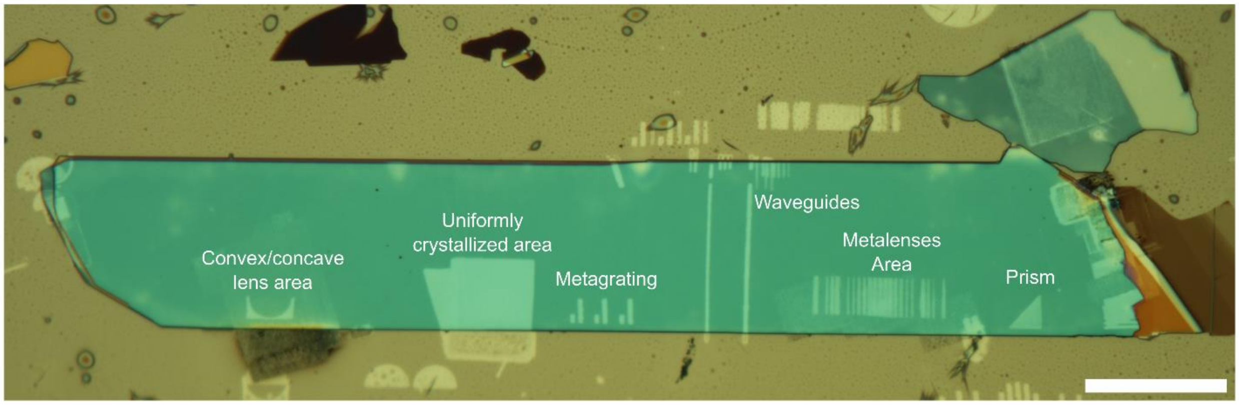

Supplementary Figure 4 | Overview picture of the hBN flake. The picture shows the flake in its final state. The flake is 220 µm long and 35 µm wide. The scale bar is 30 µm.

Supplementary Note 4: s-SNOM image processing

The images shown in the main paper have been processed to enhance the mode launched by the edge and coupled in the written structures. The raw data (collected with a commercial s-SNOM from NeaSpec, GmbH) consists of amplitude and phase images obtained via pseudo-heterodyne demodulation. The system performs the demodulation at several pseudo heterodyne harmonics, though only the first two harmonics were used in this work. The s-SNOM images are obtained by shining light on a tip scanning the sample and collecting the demodulated near field optical amplitude and phase for each pixel. Therefore, each pixel contains a complex number that represents phase and amplitude of the near field in that position. The interpretation of the s-SNOM images is not obvious in samples that can support guided modes or standing resonances as explained in our previous works[8,12,18]. When studying the propagation of guided waves, the complex s-SNOM maps are the superposition of several contributions, each due to a possible light path between the s-SNOM laser source and the detector. These dominant contributions are:

- The *material contrast contribution*, which is associated to photons interacting with the local polarizability of the material just below the tip and which are not coupled into the guided modes. This contribution exists for any sample, even when no guided modes exists.

- The *direct coupling contribution*, in which guided modes launched by edges or discontinuities propagate until they reach the s-SNOM tip, which probes them locally and scatters them to the detector. For guided modes, this contribution appears as fringes with periodicity approximately equal to the guided wavelength. The approximation comes into play because the exciting incident light is at an angle, and thus affects the periodicity of the observed fringes. This effect can be neglected in strongly confined modes.
- The *round-trip contribution*, in which the guided mode is excited by the tip, reflected by an edge or by a discontinuity and reaches back to the tip. For guided modes, this contribution also appears as fringes, but the periodicity is exactly half of the guided wavelength. This is due to the fact that polaritons propagate twice between the tip and the discontinuity.

In our work, we use the round-trip contribution to determine the effective index of the measured samples (Figure 3h) since the fringe spacing is exactly half of the guided wavelength regardless of the incident light. This provides more precise measurements than the direct coupling. For imaging the fields, we use the direct contribution instead, which provides the most faithful representation of light propagation on the sample. To excite the direct contribution, the hBN edge must be oriented perpendicularly to the light polarization, while the roundtrip contribution does not depend on the edge orientation. Therefore, the edge orientation can be used to control which of the contributions is dominant in the image. Supplementary table 1 lists the orientation of the edge and the corresponding dominant contribution for all figures in the paper. The table also lists in detail the image processing methods used for each image in the main article.

Supplementary Table 3 | Extraction of contributions in the main figures

Fig.	Edge and polarization orientation	Direct/ roundtrip	Image processing
1f	Not aligned	Both visible	Raw s-SNOM, amplitude
2i	Perpendicular	Direct	Average subtraction, vertical fringes removal, real part
2k	Perpendicular	Direct	Average subtraction, real part
2l	Perpendicular	Direct	Average subtraction, vertical fringes removal, real part
3f	Perpendicular	Direct	Average subtraction, real part
3g	Perpendicular	Direct	Average subtraction, real part
3h	Parallel	Roundtrip	Data measured from fringes in raw s-SNOM amplitude
3f	Perpendicular	Direct	Average subtraction, vertical fringes removal, real part
4g	Perpendicular	Direct	Average subtraction, vertical fringes removal, real part

The material contrast contribution is a constant complex number on all the hBN flakes and can be removed by calculating the average of the fields on the flake and subtracting this number from all pixels in the image. After that, the wavefronts can be visualized by taking the real part of the field.

For lenses we implement an additional image processing which allows to remove the vertical fringes launched by the opposite edge of the flake and to remove the fields diffracting around the lens. This is achieved by subtracting column by column the fringes launched by the edges which do not interact with the lens (see Supplementary Figure 5 for details). The result is the portion of direct contribution that is transmitted through the lens.

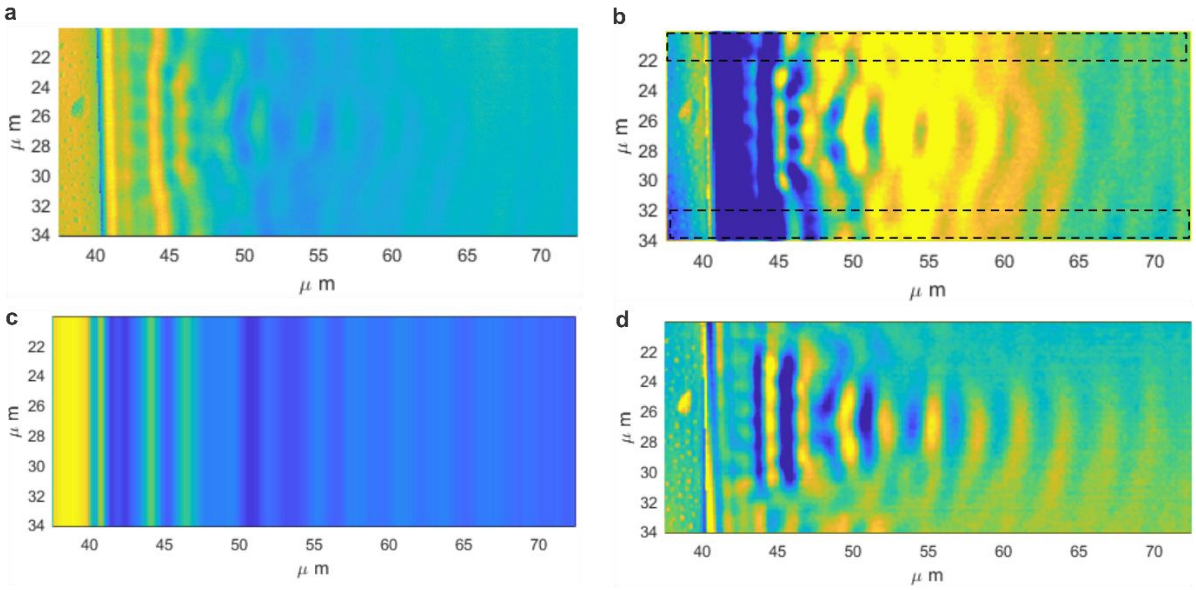

Supplementary Figure 5 | Image processing and vertical fringes removal. **a**, initial s-SNOM image (absolute value). **b**, the raw amplitude and phase maps are combined to form a complex valued map, then an average complex value (material contribution) computed on the hBN flake is removed from the image. Taking the real part gives the image shown in **b**. Taking the average of the pixel in the marginal areas above and below the lens (dashed rectangle) gives a vector of complex values, that is repeated for each row in the image represented in **c**. Subtracting map **c** from map **b** and taking the real part gives the final image in **d**.

Supplementary Note 5: Additional s-SNOM measurements

Supplementary Figures 6 and 7 illustrate the selective excitation of roundtrip and direct contributions using different polarization of the incident light. The polarization of light is not changed directly on the s-SNOM setup (it is always p-polarized), rather the sample is rotated so that the edge is either perpendicular or parallel to the in-plane projection of the polarization. The roundtrip contribution is ideally suited to precisely measure the guided wavelength, while the direct contribution partially depends on the direction of the incident wave. For the waveguide, the round-trip contribution is clearly visible away from the edge since the mode is confined by the waveguide both for the launched and reflected waves.

Supplementary Figure 8 shows s-SNOM images of a single c-GST dot written below hBN. A hotspot is clearly visible, similarly to ref. 27.

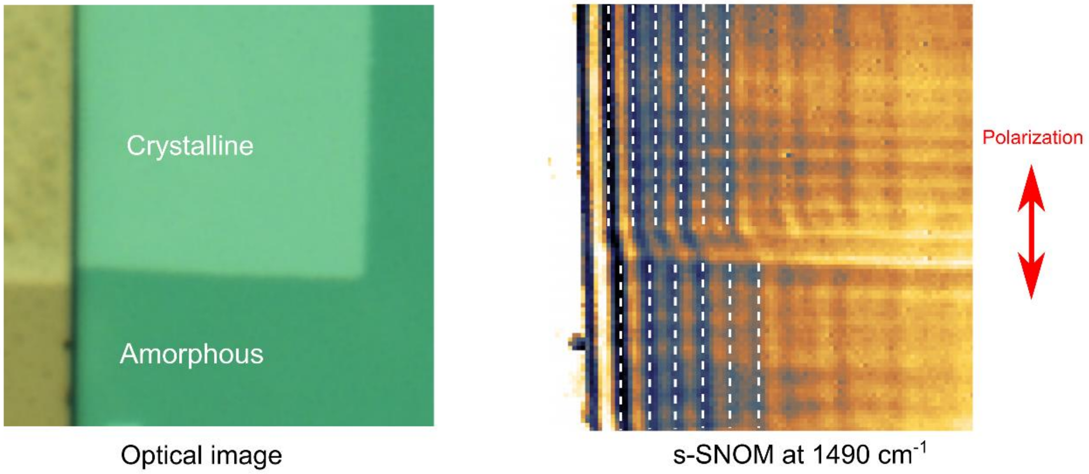

Supplementary Figure 6 | Exciting roundtrip contribution in uniformly crystallised area. The roundtrip contribution formed due to reflection of the hBN edge is visible as vertical fringes. The fringe spacing corresponds to exactly half of the guided wavelength and was used to experimentally validate the polariton dispersion. The polarization is parallel to the edge to minimize the direct contribution. A clear difference in the guided wavelength is observed in the two areas.

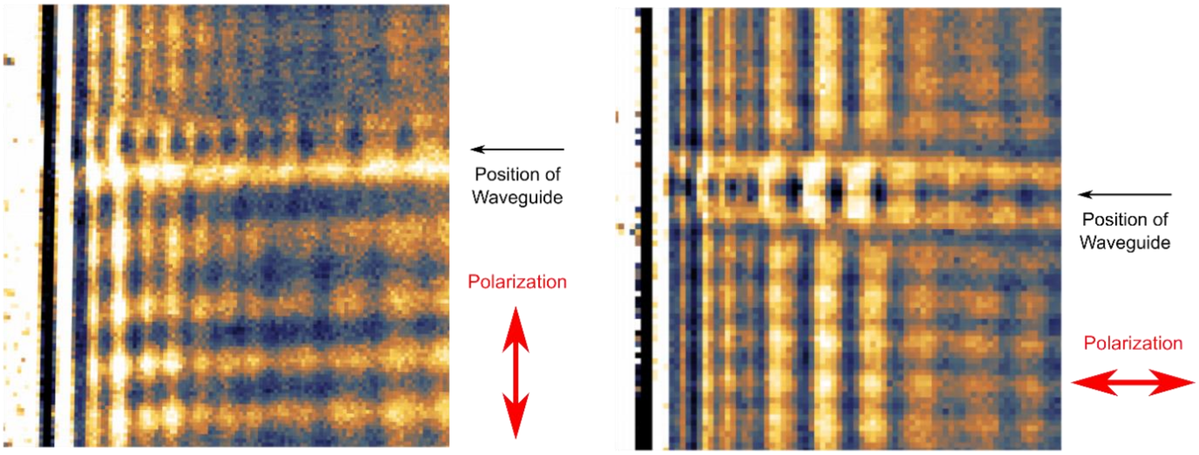

Supplementary Figure 7 | Exciting direct and roundtrip components in waveguides. Different polarization conditions were used in the waveguide structure to excite only the roundtrip contribution (polarization parallel to edge) or also the direct contribution (polarization perpendicular to the edge). s-SNOM images are taken at 1500 cm^{-1}.

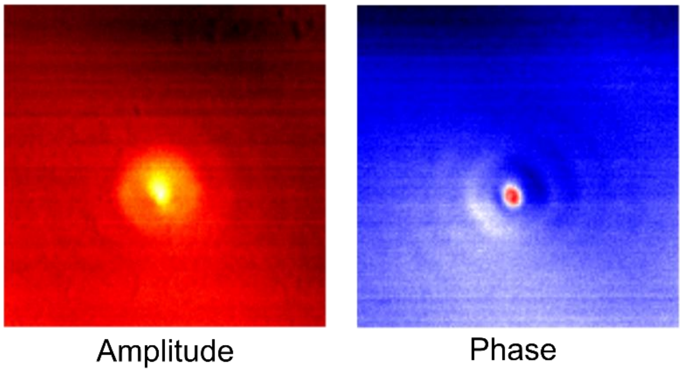

Supplementary Figure 8 | Characterisation of c-GST dot below hBN. The dot is formed by focusing the diode laser in a single position to locally crystallise GST. The dot has a diameter of approximately 800 nm, and these s-SNOM images were taken at 1500 cm^{-1} (scan area is 5 µm × 5 µm). The response is clearly visible both in the amplitude and in the phase.
