# Supplementary material for: Polariton nanophotonics using phase-change materials
Source: Nat Commun. 2019 Oct 3;10:4487. doi: 10.1038/s41467-019-12439-4 (PMC6776658; doi:10.1038/s41467-019-12439-4)
Supplement: Supplementary file 5 — Description of Additional Supplementary Files [file 41467_2019_12439_MOESM5_ESM.pdf]

Title: Supplementary Movie 1:
Description: Example of GST writing: Focused laser light (not visible due to filter) is used to write the metalens “Metalens 1”.

Title: Supplementary Movie 2:
Description: Example of GST erasing: an area of GST is written and subsequently erased

Title: Supplementary Movie 3:
Description: Animated sequence of writing and rewriting arbitrary images: each frame contains an image which is written in the same position as the previous one after erasing it.
